# Sarcoidosis misdiagnosed as malignant tumors: a case report

**DOI:** 10.1186/s12957-015-0748-6

**Published:** 2015-12-12

**Authors:** Zuosheng Li, Xin Li, Zuoqing Song, Jinghao Liu, Ming Dong, Tao Shi, Dian Ren, Song Xu, Jun Chen

**Affiliations:** Department of Lung Cancer Surgery, Lung Cancer Institute, Tianjin Medical University General Hospital, No. 154 Anshan Road, Heping District, Tianjin, 300052 China; Department of Thoracic Surgery, North China University of Science and Technology Affiliated Hospital, Tangshan, 063000 China

**Keywords:** Sarcoidosis, Lung cancer, Differential diagnosis

## Abstract

**Background:**

Sarcoidosis is a rare condition that is often misdiagnosed as malignant tumors due to the similar clinical manifestations and imaging findings.

**Case Presentation:**

We encountered a 56-year-old Chinese woman who had a chief complaint of a persistent cough. The chest computer tomography (CT) revealed mediastinal and bilateral hilar lymph node enlargement, and positron emission tomography-computer tomography (PET-CT) revealed abnormal fluorodeoxyglucose (FDG) uptake in the lymph nodes of the chest and abdomen. To further clarify the diagnosis, a lymph node sampling was performed by video-assisted thoracoscopic surgery (VATS) and the histopathologic diagnosis of sarcoidosis was confirmed.

**Conclusions:**

VATS could be an effective and minimally invasive diagnostic method to discriminate pulmonary sarcoidosis with other malignant tumors.

## Background

Sarcoidosis is an uncommon disease of unknown etiology that is characterized by the presence of noncaseating granulomas [1]. Sarcoidosis affects people of all racial and ethnic groups and occurs at all ages, although it usually develops before the age of 50 years, with the incidence peaking at 20 to 39 years [2]. Sarcoidal granulomas can involve any organ, but in more than 90 % of patients, clinical sarcoidosis is manifested as intrathoracic lymph node enlargement, pulmonary involvement, skin or ocular signs and symptoms, or some combination of these findings [3]. Systemic symptoms such as fatigue, night sweats, and weight loss are common. Sarcoidosis patients may be misdiagnosed with tuberculosis, lymphoma, or lung cancer [4]. The diagnosis of sarcoidosis is established on the basis of compatible clinical and radiologic findings, supported by histologic evidence in one or more organs of noncaseating epithelioid-cell granulomas in the absence of organisms or particles.

## Case presentation

**Fig. 1:**
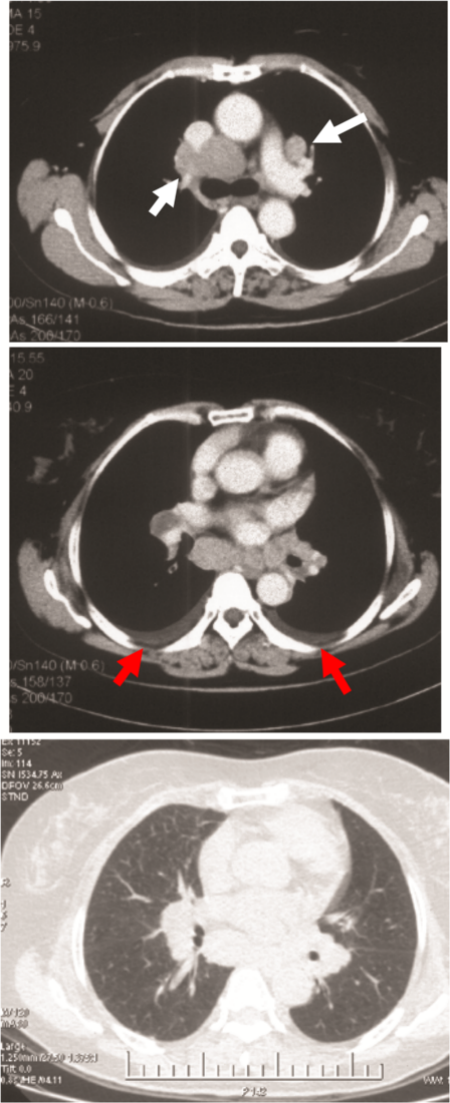
CT scans of the chest. An enhanced chest CT scans indicated a right hilar mass and multiple lymph node enlargements in the mediastinal and bilateral hilar areas of the lungs (*white arrows*), as well as bilateral small pleural effusions (*red arrows*)

**Fig. 2:**
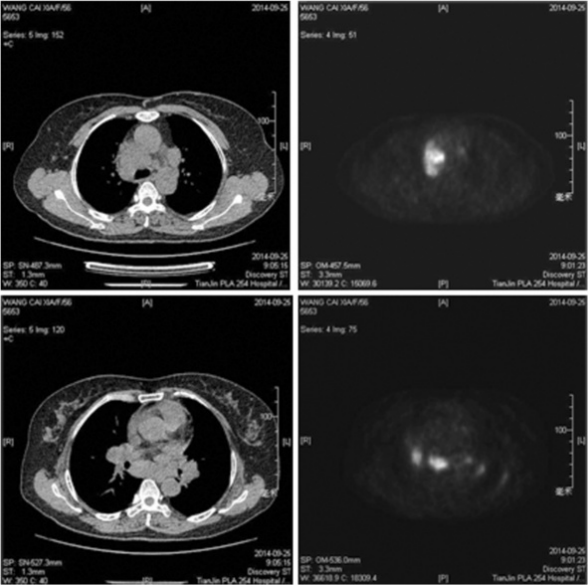
PET-CT scan. PET-CT revealed abnormal FDG uptake in the lymph nodes of the upper mediastinum and bilateral hilar areas of the lungs

A 56-year-old Chinese woman was admitted with cough and expectoration (white phlegm) for 1 month. These symptoms developed without apparent cause and in the absence of other problems, such as chest pain and/or distress, breathlessness, fever, hemoptysis, nausea, or vomiting. She denied the presence of any chronic disorders and smoking and had no family history of cancers. An enhanced chest computed tomography (CT) scan imaged a right hilar mass and a small amount of bilateral pleural effusions, accompanied with the enlargement of multiple lymph nodes in the mediastinal and bilateral hilar areas, as shown in Fig. 1. The blood tests and urinalysis on admission were within normal limits. To assist the diagnosis of the mass, a panel of tumor biomarkers, including CEA (carcino-embryonic antigen), NSE (neuron-specific enolase), SCC (squamous cell carcinoma), and CYFRA21-1 (cytokeratin 19 fragments), were tested. Only an upregulated level of NSE was observed (19.94 μg/L, normal reference value 0–16.3 μg/L). Bronchoscopic exam did not reveal any abnormality, and a transbronchial biopsy by endobronchial ultrasound guided transbronchial needle aspiration (EBUS-TBNA) did not found collections of malignant cells either. A body fluorodeoxyglucose (FDG) positron emission tomography-computer tomography (PET-CT) demonstrated remarkably increased FDG uptake in the hilus of the right lung, accompanied by multiple lymph node enlargements in bilateral hilar areas, mediastinum, and abdomen (Fig. 2). Therefore, the diagnosis of malignant tumors was preliminarily made.

**Fig. 3:**
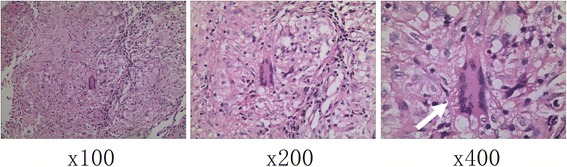
Histopathological diagnosis. The routine H&E staining proved a diagnosis of sarcoidosis with granuloma plus Langhans giant cells (*white arrows*)

To further clarify the diagnosis, a subcarinal lymph node resection was performed by right-sided video-assisted thoracoscopic (VATS). In brief, a 40-mm incision was made in the fifth intercostal space of the right anterior axillary line for the instrumental operation. A 30-degree 10 mm high definition thoracoscope was employed through the seventh intercostal space of the right mid-axillary line. It was observed from the thoracoscope that mediastinal lymph nodes were diffusely enlarged, and lymph node biopsy was applied by a harmonic scalpel. As shown in Fig. 3, histopathologic examination was granulomatous lesions and a diagnosis of sarcoidosis was confirmed. After the postoperative recovery, the patient was transferred to the respiratory department to receive further medication treatment.

### Discussion

Sarcoidosis is a multisystemic disorder of unknown etiology that is characterized by the presence of noncaseating granulomas and the proliferation of epithelioid cells [1]. Sarcoidosis can involve multiple organs; the lungs and lymph node are the most frequently involved organs. The cause and pathogenesis are unknown; however, a number of etiologies have been proposed in recent years, primarily based on genetic and immunologic factors [5]. The diagnosis of sarcoidosis is commonly established based on clinical and radiologic findings that are supported by histologic findings. The diagnosis of sarcoidosis is often delayed for various reasons, mainly due that the imaging is not suggestive sometimes. A study from Brazil evaluated 100 patients with a biopsy-proven diagnosis of sarcoidosis. The median number of physicians consulted was 3 (range, 1–14), and the diagnosis of sarcoidosis was timely in 41 patients and delayed in 59 [6]. The typical radiologic findings associated with sarcoidosis include symmetric, bilateral hilar, and paratracheal lymphadenopathy, with or without concomitant parenchymal abnormalities. However, in 25–30 % of cases, the radiologic findings are atypical, which causes difficulty in making a correct diagnosis [7]. Atypical forms of intrathoracic sarcoidosis have been described as unilateral or asymmetric lymphadenopathy, necrosis or cavitation, large opacity, ground glass opacity, airway abnormality, pleural involvement, and the reversed halo sign [7–9]. A better understanding of the radiologic manifestations of sarcoidosis is very helpful for making a correct diagnosis. In addition, we observed that NSE, a well-known tumor marker for neuroendocrine tumors, was upregulated a bit in this case. Given the radiological image and the upregulated value of NSE, the diagnosis of small cell lung cancer was ever considered before the biopsy of VATS. However, the final pathological diagnosis did not support our speculation, indicating that sarcoidosis may be misdiagnosed with other malignant tumors if only tumor markers were considered.

In recent years, PET-CT has become a superior means of diagnosis of sarcoidosis. Moreover, ^18^F-FDG PET has been proven to be a very useful imaging technique in diagnosis, disease activity assessment, monitoring treatment response, and risk assessment in patients with sarcoidosis [10]. In suspected or known sarcoidosis patients, PET-CT may be useful in the evaluation of disease extent and monitoring treatment response [11]. In atypical, complex, and multisystem sarcoidosis, PET-CT plays an important role for the evaluation of the localization and effect of treatment [12]. Although it has so many advantages, PET-CT can cause many false-positive and its role in the diagnosis of sarcoidosis is limited [13]. Pleural effusion is rare in patients with pulmonary sarcoidosis. Only about 2.8–5 % of these patients exhibit pleural effusion [14–16]. A pleural effusion in patients with sarcoidosis should not be assumed to be related to sarcoidosis [16]. Pleural effusion in sarcoidosis is often an exudate. The most common cause of exudative pleural effusion in elderly patients is a malignant tumor or tuberculosis. Sarcoidosis is hard to be differentiated with the two diseases. In this patient, the CT scan showed a right hilar mass and a small amount of bilateral pleural effusions, accompanied with the enlargement of multiple lymph nodes in the mediastinal and bilateral hilar areas, while PET-CT indicated a high FDG uptake in these lesions. The pathological diagnosis of sarcoidosis was determined after a video-assisted thoracoscopic procedure. Timely and correct diagnosis is very important for improving pulmonary function and determining the presence of lesions in other organs, while delayed diagnosis is often associated with an impaired lung function and medication induced impairment of the other organs [6]. For example, many sarcoidosis patients are misdiagnosed with tuberculosis and treated with anti-tuberculosis drugs who eventually developed with liver function impairment. In addition, some serious forms of sarcoidosis, such as cardiac sarcoidosis, can appear undetected because of the lack of systematic investigation [6]. For these reasons, the diagnosis of sarcoidosis should be promptly determined in order to avoid treatment delay. Surgical intervention is an important modality for the attainment of a biopsy. VATS could be used as a valid minimally invasive method for the diagnosis of sarcoidosis, as it allows larger samples of tissue and greater diagnostic yield. Furthermore, in comparison with the traditional open techniques, VATS offers less postoperative pain, a more rapid recovery, and much less complications [17, 18].

## Conclusions

In conclusion, sarcoidosis is difficult to be differentiated with other malignant tumors by radiological and PET-CT examinations. VATS biopsy is a minimally invasive, safe, and effective procedure in the diagnosis of pulmonary sarcoidosis.

### Consent

The patient granted written informed consent for publication of this manuscript and the accompanying images. A copy of the written consent is available for review by the Editor-in-Chief of this journal.

### Institutional review board statement

This material has not been published and is not under consideration elsewhere. There is no financial disclosure from each author.
